# Advanced Radiation Techniques in the Treatment of Esthesioneuroblastoma: A 7-Year Single-Institution’s Clinical Experience

**DOI:** 10.3390/cancers10110457

**Published:** 2018-11-20

**Authors:** Jakob Liermann, Mustafa Syed, Thomas Held, Denise Bernhardt, Peter Plinkert, Christine Jungk, Andreas Unterberg, Stefan Rieken, Jürgen Debus, Klaus Herfarth, Sebastian Adeberg

**Affiliations:** 1 Department of Radiation Oncology, Heidelberg University Hospital, 69120 Heidelberg, Germany; jakob.liermann@med.uni-heidelberg.de (J.L.); mustafa.syed@med.uni-heidelberg.de (M.S.); thomas.held@med.uni-heidelberg.de (T.H.); denise.bernhardt@med.uni-heidelberg.de (D.B.); stefan.rieken@med.uni-heidelberg.de (S.R.); juergen.debus@med.uni-heidelberg.de (J.D.); klaus.herfarth@med.uni-heidelberg.de (K.H.); 2 Heidelberg Institute of Radiation Oncology (HIRO), 69120 Heidelberg, Germany; 3 Heidelberg Ion-Beam Therapy Center (HIT), Department of Radiation Oncology, Heidelberg University Hospital, 69120 Heidelberg, Germany; 4 National Center for Tumor Diseases (NCT), 69120 Heidelberg, Germany; 5 Clinical Cooperation Unit Radiation Oncology, German Cancer Research Center (DKFZ), 69120 Heidelberg, Germany; 6 German Cancer Consortium (DKTK), Partner Site, 69120 Heidelberg, Germany; 7 Department of Otolaryngology, Head and Neck Surgery, Heidelberg University Hospital, 69120 Heidelberg, Germany; peter_plinkert@med.uni-heidelberg.de; 8 Division of Neurosurgical Research, Department of Neurosurgery, Heidelberg University Hospital, 69120 Heidelberg, Germany; christine.jungk@med.uni-heidelberg.de (C.J.); andreas.unterberg@med.uni-heidelberg.de (A.U.)

**Keywords:** esthesioneuroblastoma, olfactory neuroblastoma, radiotherapy, particle therapy, carbon ion radiation

## Abstract

(1) Background: Esthesioneuroblastoma (ENB) is a rare tumor entity originating from the olfactory neuroepithelium. There is a scarcity of data about different treatment strategies. Intensity modulated radiotherapy (IMRT) and carbon ion radiotherapy (CIRT) are advanced radiation techniques that might improve local tumor control. (2) Methods: This retrospective analysis contained 17 patients with ENB (Kadish stage ≥ C: 88%; *n* = 15). Four patients had already undergone previous radiotherapy (RT). The treatment consisted of either IMRT (*n* = 5), CIRT (*n* = 4) or a combination of both techniques (*n* = 8). Median follow-up was 29 months. (3) Results: In patients that had not been irradiated before (*n* = 13), calculated overall survival (OS) and progression free survival (PFS) rates after 48 months were 100% and 81% respectively (Kaplan-Meier estimates). Two of four patients that underwent reirradiation died after RT, presumably due to tumor progression. Besides common toxicities, five patients (30%) showed mostly asymptomatic radiation-induced brain changes, most likely due to a disturbance of the blood-brain barrier. (4) Conclusions: Our results demonstrate that IMRT, CIRT, a combined approach of IMRT and CIRT as well as reirradiation with CIRT seem to be feasible and effective treatment methods in ENB.

## 1. Introduction

Esthesioneuroblastoma (ENB) or olfactory neuroblastoma is a rare tumor entity that seems to originate from the olfactory neuroepithelium. Only 3–6% of all cancers of the nasal cavity or paranasal sinuses can be categorized as ENB [1,2,3]. It was first described by Berger et al. in 1924 [4] but still most of the published data rely on small cohorts due to the rarity of this tumor. Men and women are affected equally. The mean age of initial diagnosis is thought to be at 40–70 years [5]. The incidence seems to have increased in the last decade, underlining the necessity for finding appropriate, well-designed and tumor-specific treatment options [6]. Still, prospective data remain illusive due to the limited number of patients. 

The tumors are classified by a staging system established by Kadish et al. in 1976, which was modified in 1993 by Morita et al. [7,8]. The 10-year overall survival (OS) rate of patients with a Kadish stage A tumor is reported to be 83%, decreasing to only 13.3% in Kadish stage D tumors [5]. In terms of histology, Hyams et al. described a grading score that correlates with worse prognosis based on degree of tissue necrosis and mitotic activity [2,9,10,11].

Due to a lack of prospective randomized studies, it still remains uncertain which treatment option is the best. Radiotherapy should only be postponed in Kadish stage A tumors if a complete resection could be achieved. Generally, a craniofacial approach seems to be an advantage for most of the tumors [2]. Still, endoscopic surgery is shown to be effective [12]. Given the typical localization of the tumor, a complete resection, especially in Kadish stage B or higher staged tumors, is difficult and rarely achievable. In these cases, additive radiotherapy (RT) should be performed, as multimodal treatment has been shown to be more effective [5,13,14]. RT alone is an alternative to surgery in all stages but seems to be less effective than a multimodal approach [15]. The use of chemotherapy in the treatment of ENB still remains unclear [2,13].

**Figure 1:**
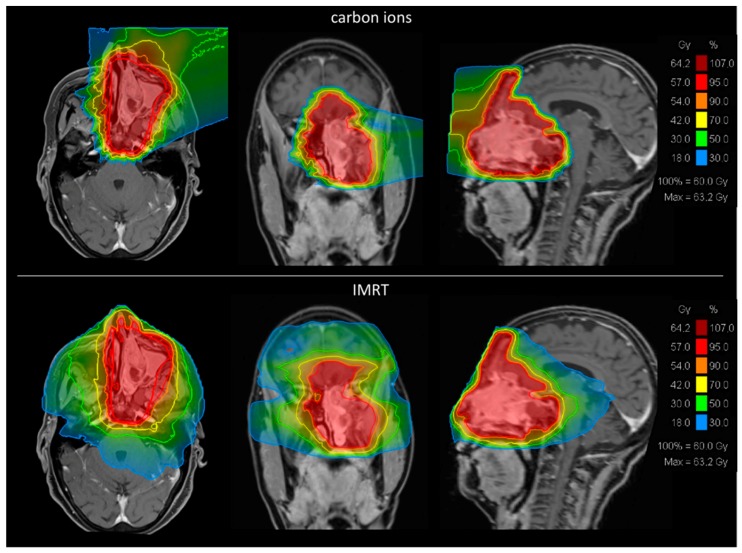
Representative comparison of carbon ion radiotherapy (CIRT) and intensity modulated radiotherapy (IMRT) plans to show general differences. Comparing radiation plans of CIRT and IMRT, carbon ion radiation shows a remarkable higher dose conformity than photon irradiation.

Irradiation has to be very precise because of the delicate localization of the tumor. Critical organs at risk (OAR) like the optical system, the frontal and temporal lobes, the brainstem and the pituitary gland are usually located adjacent to the tumor. Moreover, radiation dose has to be adequately high to improve local tumor control [16,17]. Therefore, precise radiation techniques with an advanced dose conformity, such as intensity modulated radiotherapy (IMRT) or particle therapy, are needed [18]. Particle therapy is even reported to lead to better outcomes than IMRT in patients with tumors of the nasal cavity [19] presumably due to its greater dose conformity and higher biological efficiency. To demonstrate this principle, we calculated two representative radiation plans with IMRT and carbon ion radiotherapy (CIRT) for one patient with ENB (Figure 1).

In 1993, Foote et al. recommended to irradiate ENBs with a dose of 55.0 Gy (single dose 2.0 Gy) [16]. Ozsahin et al. described an impact on survival when a dose higher than 54.0 Gy is applied [20]. To further reduce local recurrence, dose escalation should be done whenever feasible [17]. Applied as a separate boost radiation, particle therapy could be used to escalate the dosage as it has already been effective in other malignancies, e.g., adenoid-cystic carcinomas [21].

As a result of the extreme rarity of these tumors, only a few studies have been published so far. During 2010–2016, we irradiated 17 patients with ENB in our institution. Here, we present our experience using different radiation techniques in different clinical settings.

## 2. Results

### 2.1. Group A (First Irradiation, 13 Patients)

**Figure 2:**
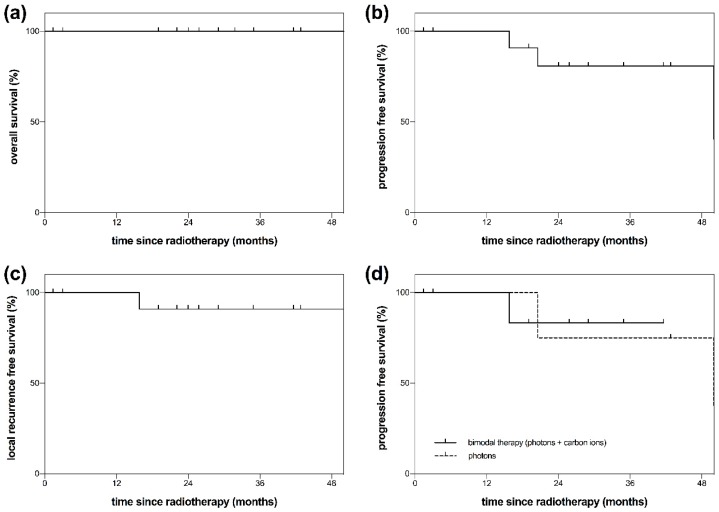
Overall survival (OS), progression free survival (PFS) and local recurrence free survival (LRFS) of patients with esthesioneuroblastoma (ENB). The presented patients (*n* = 13) had not been irradiated before (group A). (**a**) OS of patients with ENB after radiotherapy (RT) (group A). (**b**) PFS of patients with ENB (group A). Progression was defined as both local or distant tumor recurrence. (**c**) LRFS of patients with ENB (group A). Local recurrence was defined as tumor recurrence at the primary tumor site (local failure). Regional failure was not included. (**d**) PFS of patients with ENB after RT (group A). Comparison between photons and a bimodal radiation technique consisting of photons plus carbon ion boost radiation. PFS differences could not be calculated due to the limited number of events.

Group A consisted of 13 patients that had not been irradiated before. After a median follow-up of 29 months (range: 1–95) all patients were still alive to the best of our clinical knowledge (Figure 2a). Tumor progression could be observed in three patients. The median PFS was 65 months (range: 1–95 months). The PFS rate after 48 months was 81% (Figure 2b). After 72 months, a PFS rate of 40% could be observed. The LRFS rate was 91% after 48 months (Figure 2c). Tumor progression could be observed after bimodal RT, consisting of IMRT + CIRT (*n* = 1), and after photon radiation only (*n* = 2). PFS differences between photon radiation and the bimodal approach (IMRT + CIRT) could not be calculated due to the limited number of events (Figure 2d).

### 2.2. Group B (Reirradiation, Four Patients)

Group B consisted of four patients that had undergone radiation before. The median OS of these four patients was 64 months (range: 10–64 months). Two patients died 10 and 64 months after the treatment, presumably due to tumor progression.

Another patient developed local tumor recurrence 33 months after the treatment. In total, the median PFS was 33 months (range: 6–33 months) after reirradiation.

### 2.3. Treatment Toxicity

In all patients, the treatment course could be completed as prescribed. In total, 65% of all patients developed stomatitis during RT (CTCAE grade 1–2) and 76% suffered from radiation dermatitis (CTCAE grade 1–2). Further common acute toxicities were radiation conjunctivitis, dysgeusia and headaches. Stomatitis and radiation dermatitis decreased over time and could not be observed after 12 months of follow-up. Late toxicities occurred in the form of increased mucous production, partially with the appearance of crusts (31%), xerostomia (23%), dysgeusia (38%) and anosmia (69%). In total, 23% of the patients suffered from persisting moderate fatigue 12 months after RT. Apart from anosmia no CTCAE grade 3–4 toxicity occurred.

However, in five patients (30%) new radiation-induced brain changes with contrast agent enhancement could be observed in the course of follow-up. In two cases (12%), these lesions were symptomatic. One of these two patients presented several weeks after reirradiation with an increasing contrast agent enhancement next to the resection cavity and in the radiation field with a clinical presentation of headache. Surgery was planned but the patient died before the procedure. The second patient with symptomatic brain changes presented with headache, too. He initially did not respond to corticosteroid treatment leading to the hypothesis of tumor recurrence. He was treated with a tyrosine kinase inhibitor but in the course of therapy he developed an intracerebral abscess that was operated successfully. In both cases, tumor progression was the most likely explanation but radiation necrosis could not be excluded.

**Figure 3:**
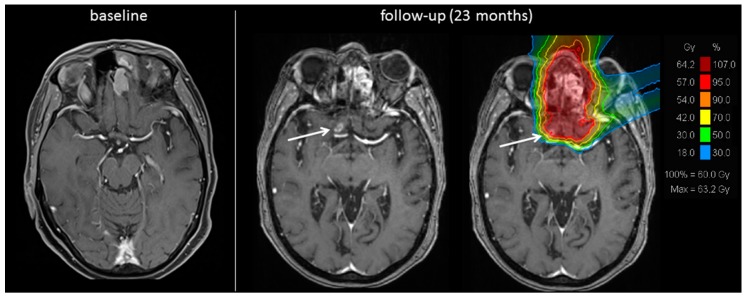
Radiation-induced cerebral contrast agent enhancement. Baseline and follow-up MRI scan of a patient with esthesioneuroblastoma (ENB). 23 months after radiotherapy a new cerebral contrast agent enhancement (white arrow) could be detected. In comparison with the initially treated radiation plan (MRI scan on the right) the lesion occurred in an irradiated region of the right frontal lobe at the edge of the 95%-isodose representing 57 Gy (RBE, 71.25 Gy EQD2). A radiation-induced disturbance of the blood-brain barrier or even radiation necrosis is probable.

In other cases, radiation-induced brain changes can be diagnosed more precisely, e.g., because of the typical localization or the distance to the primary tumor site making the lesions less likely to be a morphological correlate of tumor recurrence (Figure 3). Additional MRI sequences such as diffusion and perfusion were partially generated but did not help in diagnosis, in these cases.

The three cases of asymptomatic radiation induced brain changes have been observed in follow-up MRI imaging. If deemed necessary, the asymptomatic lesions were treated with corticosteroids or as second line therapy with bevacizumab. Some of the patients still receive ongoing treatment.

All radiation-induced brain changes were located in the frontal lobes. They were either in the radiation field or at least at the edge of the radiation field. No radiation-induced brain change could be detected beyond the radiation field and none could be seen after photon irradiation only.

## 3. Discussion

Being such a rare malignancy, clear treatment strategies of ENB are still lacking. A multimodal approach of surgery and RT is thought to be most effective but it is still unknown which radiation technique should be performed. In the presented study, we present our experience with different radiation techniques in different clinical settings. The current study has some clear limitations. The observed patient cohort consists of only 17 patients, which makes reliable findings difficult. Furthermore, the presented cases were very heterogenous. Still, considering the rarity of this tumor, we present a substantial cohort of patients undergoing modern radiation techniques. Most of the published cases are described in case studies or a single-institution’s retrospective analyses with small cohorts. To our knowledge, the biggest cohorts describing particle therapy in the treatment of ENB consist of 42 patients treated with protons from 1999 to 2012 [22] and 21 patients treated with carbon ions from 2003 to 2014 [23]. Generally, the biggest patient cohorts are known from database analyses, in which different radiation techniques are usually not further described, for example, recently in a National Cancer Database analysis investigating 931 cases of ENB [13]. To our knowledge, no prospective randomized clinical trial of patients with ENB has been published so far. Concerning radiotherapy, there is an ongoing multicenter prospective registry study in Japan that is collecting information of patients irradiated with carbon ion doses of 57.6–64.0 Gy (RBE) [23].

Our presented patient cohort shows a median age of 53 years which is the typical age for first diagnosis of ENB [5] but the presented observed sex proportion is unbalanced towards men. The median follow-up of only 29 months seems to be relatively short but is due to the original goal of this study to investigate modern radiation techniques. The treatment with carbon ions at Heidelberg Ion-Beam Therapy Center (HIT) started in 2009.

The observed OS rate of 100% in group A (first irradiation) seems to be very promising but has to be put into the perspective of the relatively short follow-up interval. Nevertheless, the presented cases were mostly locally advanced tumors (modified Kadish stage ≥ C: 88%) in which a five-year OS rate from about 50 to 77% is assumed [13,24]. The presented data, although of short interval follow-up, demonstrate a very promising overall survival rate, especially in this high-risk population.

The observed PFS of 81% after a follow-up of 48 months is in line with published data assuming it to be 73–86% after 2 years [9]. A treatment with CIRT should lead to even better local control. Three years after CIRT, Suefuji et al. observed a PFS of 83% including locally advanced tumors (T4 N0 M0) and even included recurrent tumors [23]. In the presented data, there was only one patient in group A who developed tumor progression after having received CIRT or bimodal RT (consisting of IMRT and a separate carbon ion boost irradiation), but the presented cohort is too small to analyze valid differences between IMRT only and CIRT/bimodal RT. Our data demonstrate that a bimodal concept of IMRT + CIRT seems to be an effective dosage concept.

The four patients of group B that underwent reirradiation show a much worse course of disease with two known deaths. Interpreting these results, one has to consider that there is no standard treatment available for inoperable recurrent tumors. Reirradiation is the last treatment option to gain local tumor control in most cases. Considering this, a median PFS of 33 months is still an acceptable goal. The feasibility and outcomes of reirradiation with CIRT in head and neck cancers are currently under evaluation in our institution and will be published soon.

The observed Grade 1–2 toxicities are common radiation induced side effects and can be explained by the localization of the tumor. Additionally, anosmia—being of grade 3 toxicity—is an expected toxicity considering the origin of this tumor entity. The most remarkable observed side effect is the appearance of radiation-induced brain changes that could be seen in 30% of the patients. In the bimodal RT concept of either 54 Gy photons + 18 Gy (RBE) carbon ions or 50 Gy photons + 24 Gy (RBE) carbon ions, the cumulative dose with an alpha/beta-ratio of 2 is consequently 76.5–80.0 Gy (linear quadratic equivalent dose at 2 Gy per fraction: EQD2). Schlampp et al. observed similar radiation induced side effects after CIRT in patients with skull base chordomas and chondrosarcomas. The range of contrast agent enhancement in the temporal lobes lasted from 8% of patients receiving 75 Gy (EQD2) to 50% of patients receiving 87.5 Gy (EQD2) [25]. The observed frequency of radiation-induced brain changes in the presented data is generally in line with these findings, although Schlampp et al. observed mostly asymptomatic reactions and evaluated patients that underwent CIRT only. In the presented data, we observed a higher percentage of symptomatic lesions. Furthermore, the lesions were situated in the frontal lobes. The difference in the localization can be explained by the different radiation fields when treating skull base tumors compared to ENB. The underlying biological mechanism is thought to be a disturbance of the blood-brain barrier that could even lead to cerebral radiation necrosis [26,27]. It is very difficult and often impossible to distinguish between this radiation-induced side effect and local tumor recurrence because of similar signs in radiological imaging [28]. In some of the radiation-induced brain changes, a therapy with corticosteroids seems effective. Otherwise, a therapy with bevacizumab should be considered [29]. Still, there are severe cases in which a neurosurgical approach is necessary. In one of the presented cases with suspicion of tumor progression or less likely symptomatic radiation necrosis, an operation was planned but could not be performed anymore, as mentioned above. Suefuji et al. did not report on MRI imaging as part of the follow-up in their study. Thus, they did not describe any appearance of radiation-induced brain changes after CIRT in ENB, although central nerve system necrosis was evaluated [23]. Suefuji et al. irradiated with a total dose of 65.0 Gy (RBE) in 26 fractions and up to 70.2 Gy (RBE) in 26 fractions. Single doses were 2.5–2.7 Gy (RBE), applied five times per week. In the presented data, only one patient received CIRT only. In this case, we applied 60 Gy (RBE) in 20 fractions with a single dose of 3.0 Gy (RBE), applied six times per week. A comparison of the dosimetry in different ion beam centers is extremely difficult because of the different methods of calculating it. For example, differences up to conversion factors of 0.4–2.0 can be seen when comparing the Local-effect-model (LEM), which was used in the present study, with the approach of the Heavy-Ion Medical Accelerator (HIMAC) in Chiba in Japan [30].

## 4. Materials and Methods

### 4.1. Patients’ Characteristics

We retrospectively analyzed 17 patients with histologically confirmed diagnosis of ENB who underwent RT at our institution from 2010 to 2016 (12 male, 5 female). The median follow-up was 29 months (range: 1–95 months). In 11 of the 17 patients, the performed radiation was part of the initial tumor treatment as an additive therapy after surgical resection. In another patient, RT was done because of tumor recurrence 23 years after the initial treatment by resection. Furthermore, in one patient the radiation was performed as a definitive therapy without surgical intervention. One patient was treated with concomitant chemotherapy with cisplatin because of having presented with a histologically combined diagnosis of SNUC and ENB. All other patients did not receive concomitant chemotherapy. Two of these patients presented initially with regional lymph node metastases. Distant metastases were not observed. Altogether, these 13 patients are defined as group A (first irradiation group).

The remaining 4 of the 17 patients presented in a recurrent disease stage and underwent reirradiation at our institution. They are described separately and therefore defined as group B (reirradiation group). Two of them initially presented with regional lymph node metastases.

The median age of all patients was 53 years (range: 31–77 years), the median Karnofsky performance score was 90 (range: 70–90). 88% of the patients presented with a modified Kadish stage ≥ C (*n* = 15). As part of the initial tumor treatment (*n* = 12), RT started within 3.5 months (range: 2–8 months) after tumor diagnosis. Resection status was evaluated based on operation reports, histology and postoperative imaging. Patients’ main characteristics are listed in Table 1. The study was approved by the ethics committee of the University of Heidelberg, Germany (S-421/2015).

### 4.2. Pre-Treatment Imaging

As part of the radiation planning, all patients underwent a native high-resolution computer tomography (CT) scan at our institution with a slice thickness of 3 mm and were immobilized with a thermoplastic mask. If there were no contraindications, an additional MRI scan with contrast agent (gadolinium, T1-weighted, fat-saturated if necessary, slice thickness of 3–5 mm) was performed and both MRI and CT images were matched to improve radiation planning. The gross tumor volume (GTV) was defined as visible tumor demarked by contrast agent enhancement or clear signs of tumor infiltration. The clinical target volume (CTV) was defined as assumable microscopic tumor extension and had to be clinically reasonable, especially respecting anatomic boundaries. Safety margins in the form of the planning target volume (PTV) were added according to the treatment modality (2–3 mm). The radiation dose was prescribed to the CTV. Reduced PTV coverage was partially tolerated to reduce the radiation dose in OAR. In patients receiving bimodal radiotherapy, the CTV of the boost plan was strictly reduced to the GTV with relatively small CTV and PTV margins (5–6 mm plus additional 2–3 mm). The CTV of the base plan (and in unimodal techniques the general CTV) was defined more generously, e.g., including whole paranasal sinuses in the case of bone infiltration of the sinuses’ walls.

### 4.3. Treatment Modalities–Group A (First Irradiation, 13 Patients)

Group A consists of 13 patients, that had not been irradiated before. In one patient of group A, no surgical treatment had been done. This patient was irradiated with carbon ions only with a total dose of 60 Gy (RBE) (single dose 3 Gy (RBE), alpha/beta = 2, linear quadratic equivalent dose at 2 Gy per fraction (EQD2) 75 Gy, 3D-planned, image-guided particle therapy, carbon ions, active rasterscanning, one fraction per day, 6 fractions per week, CTV 200 ccm). Another patient had received the first tumor diagnosis 23 years ago.

In the remaining 11 patients, radiotherapy followed tumor resection after first diagnosis. A complete tumor resection had only been achieved in three patients (R0). In 8 of these 11 patients, additive radiation was performed as a bimodal concept consisting of a combination of photon radiation (3D-planned, image-guided IMRT, one fraction per day, 5 fractions per week) and a separate carbon ion boost (3D-planned, image-guided particle therapy, carbon ions, active rasterscanning, alpha/beta = 2, one fraction per day, 6 fractions per week). This bimodal concept should lead to dose escalation with still acceptable radiation doses in the OAR. The total dose was 50–56 Gy photons (single dose 2 Gy) and 18–24 Gy (RBE) carbon ions (single dose 3 Gy (RBE)). The cumulative dose was 76.5–80 Gy (EQD2). Two of these patients initially presented with cervical lymph node metastases (N1), hence the cervical lymph node regions were included in the clinical target volume. The remaining 4 patients had been treated with photons only with a total dose of 60 Gy (single dose 2 Gy, 3D-planned, image-guided IMRT, one fraction per day, 5 fractions per week). The median CTV of photon radiation was 231 ccm (range: 176–542 ccm). The median CTV of the carbon ion boost radiation consisted of 111 ccm (range: 51–303 ccm).

### 4.4. Treatment Modalities–Group B (Reirradiation, Four Patients)

Group B consists of 4 patients, that had undergone radiation before. One patient was reirradiated with photons (total dose 60 Gy, single dose 2 Gy, 3D-planned, image-guided IMRT, one fraction per day, 5 fractions per week, PTV 121 ccm) after having been irradiated with 64.8 Gy photons (incl. IMRT boost irradiation) 7.5 years earlier. The reirradiation of the remaining 3 patients consisted of a reirradiation with CIRT (reCIRT) with a total dose of 45–51 Gy (RBE) (single dose 3 Gy (RBE), alpha/beta = 2, 56.25–63.75 Gy (EQD2), 3D-planned, image-guided particle therapy, carbon ions, active rasterscanning, one fraction per day, 6 fractions per week) after photon irradiations with 50.4–55.8 Gy 3.5, 8 and 12 years before. The median CTV was 338 ccm (range: 227–551 ccm).

### 4.5. Follow-Up

Follow-up consisted of MRI imaging every three months within the first two years after completion of RT, as well as regular clinical examinations to evaluate outcome and potential tumor progression. At 3–5 years after RT, the frequency of MRI imaging and clinical examinations was performed in 6-month intervals. Two patients were lost to follow-up. The median follow-up was 29 months (range: 1–95 months).

### 4.6. Overall Survival (OS)

Median OS and OS rates were calculated by Kaplan-Meier estimates. The observed time was defined as start of RT until death or the last follow-up. Every known death was counted as an event. Patients alive and patients lost to follow-up were counted as censored.

### 4.7. Progression Free Survival (PFS)

Median PFS and PFS rates were calculated by Kaplan-Meier estimates. The observed time was defined as start of RT until tumor progression/death or the last follow-up. Local or distant tumor progression were counted as events. Patients without tumor progression and patients lost to follow-up were counted as censored.

### 4.8. Local Recurrence Free Survival (LRFS)

LRFS rates were calculated by Kaplan-Meier estimates. The observed time was defined as start of RT until local tumor progression at the primary tumor site or last follow-up. Metastases were defined as distant recurrence and were not counted as events, therefore LRFS did not include regional failure. Patients without local recurrence and patients lost to follow-up were counted as censored.

### 4.9. Treatment Toxicity

Acute toxicity was evaluated at the end of RT. Late toxicity was evaluated 6–8 weeks and 12 months after completion of radiation and was described according to the Common Terminology Criteria for Adverse Events (CTCAE) criteria (version 4.03). 

### 4.10. Statistical Analysis

For statistical analysis, Kaplan-Meier estimates were calculated. Statistics and figures were performed with GraphPad Prism 7 for Mac (GraphPad Software, La Jolla, CA, USA).

## 5. Conclusions

The current data demonstrate different radiation techniques in the treatment of ENB. Considering the advanced tumor stage of the cohort, the results show acceptable PFS and OS rates in short-term follow-ups. IMRT, CIRT, a combined treatment of IMRT and CIRT as well as reCIRT seem to be feasible and effective approaches in ENB, that merit further evaluation.

## Figures and Tables

**Table 1:** Main patients’ characteristics.

Characteristics	No of Patients
Gender	
Male	12
Female	5
Kadish stage (modified)	
A	0
B	2
C	11
D	4
Hyams-Grading	
1	1
2	6
3	2
4	0
not done	8
Surgery	
preceding resection	14
no preceding resection	3
R0	3
R1	1
R2	9
RX	1
irradiation	
first irradiation	13
reirradiation	4
photons	5
carbon ions	4
photons + carbon ions	8
